# Supplementary material for: Integrated Design Fabrication and Control of a Bioinspired Multimaterial Soft Robotic Hand
Source: Cyborg Bionic Syst. 2023 Aug 8;4:0051. doi: 10.34133/cbsystems.0051 (PMC10408382; doi:10.34133/cbsystems.0051)
Supplement: Supplementary 1 — Fig. S1 Tables S1 and S2 Movies S1 to S3 [file cbsystems.0051.f1.zip › SM.docx]

Supplementary Materials

Figure S1

Tables S1 to S2

Movies S1 to S3


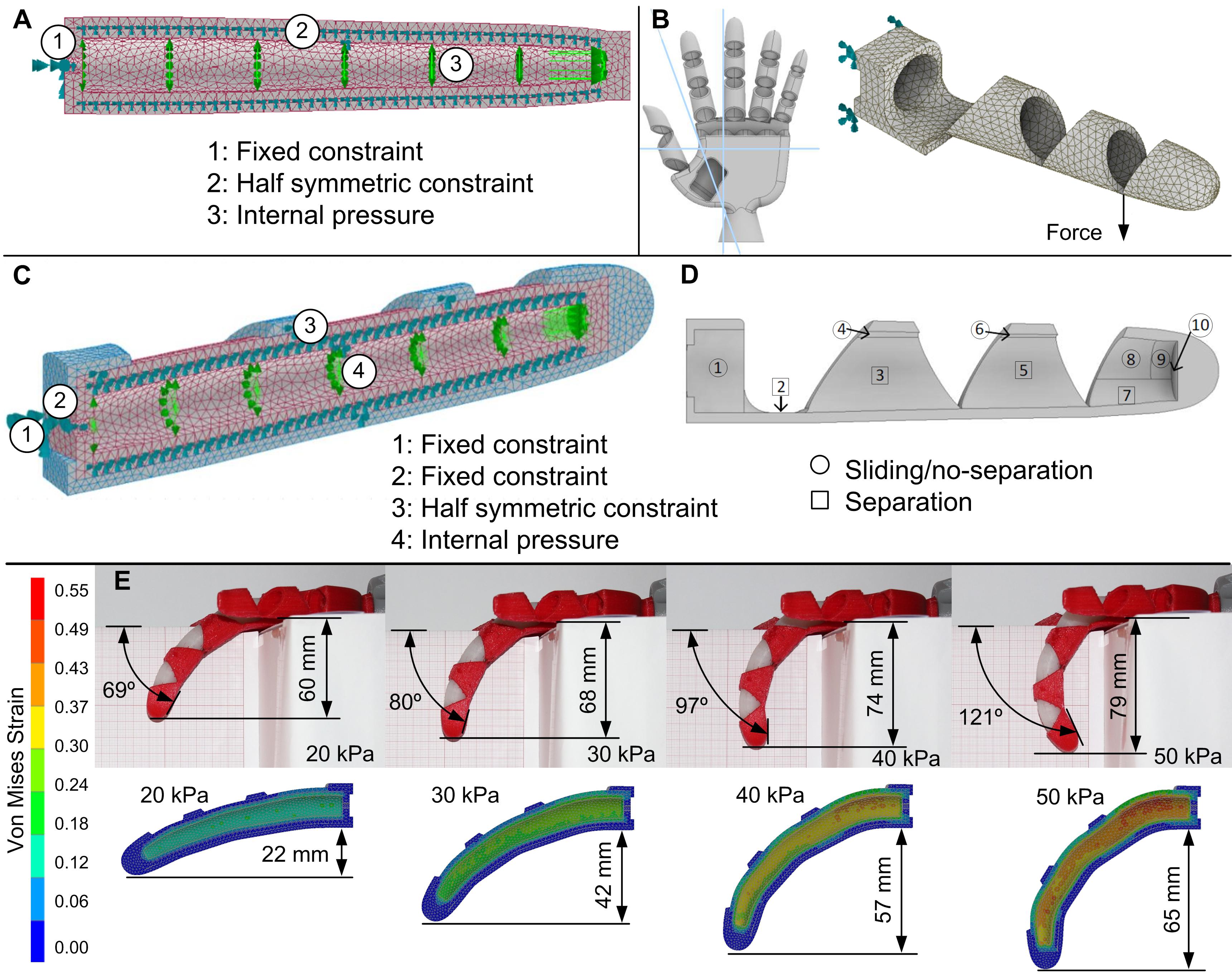


**Fig. S1**. FEA finger’s constraints. (A) Applied constraints to the actuator, which include a fixed constraint on the base, a half-symmetric constraint, and the internal pressure on the chamber walls. (B) Exoskeleton FEA mesh. Each exoskeleton finger is fixed on the base and the load force applied at the distal joint. (C) Applied constraints to the complete finger. (D) Sliding/separation areas between the actuator and the exoskeleton. (E) Numerical and experimental configuration of the complete finger for different values of pressure, highlighting the strain values inside the actuator chamber.

**Table S1**. Three different sets of material parameters of the Ogden hyper-elastic model are used to describe the platinum-catalyzed silicone.

| Set | Parameters | | Incompressible value |
| --- | --- | --- | --- |
| Ogden set1 | α_1_=1.55  α_2_=7.86  α_3_=-1.91 | μ_1_=107900.00  μ _2_=21.47  μ _3_=-87100.00 | D_1_=1x10^5^ |
| Ogden set2 | α_1_=1.05  α_2_=4.00  α_3_=-1.60 | μ_1_=1.50 x10^5^  μ _2_=60.00  μ _3_=-1300.00 | D_1_=1x10^5^ |
| Ogden set3 | α_1_=1.05  α_2_=4.00  α_3_=-1.60 | μ_1_=1.12 x10^5^  μ _2_=45.00  μ _3_=-975.00 | D_1_=1x10^5^ |

**Table S2**. Three different sets of material parameters of the MR hyper-elastic model are used to describe thermoplastic polyurethane.

| Set | Parameters [MPa] | | Material density [g/cm^3^] |
| --- | --- | --- | --- |
| MR set1 | C_10_=0.677 | C_01_=1.621 | 1.19 |
| MR set2 | C_10_=0.300 | C_01_=0.750 | 0.83 |
| MR set3 | C_10_=0.210 | C_01_=0.525 | 0.83 |

**Movie S1**. Object Grasping and Manipulation

The soft robotic hand is attached to a robot manipulator (MOTOMAN SIA, Yaskawa, Japan), grasping and manipulating different objects from the Yale-CMU-Berkeley (YCB) dataset. It successfully grasps and manipulates the objects (fruits models and plastic/ceramic cups) at a robot speed of 100 mm/s with no visible slippage.

**Movie S2**. Fabrication

The fabrication of the soft robotic hand follows a sequential process, starting from the CAD models of the exoskeleton and actuators to their fabrication. The exoskeleton is 3D printed in a single part using FFF. The fabrication of the actuators using molding follows a sequential process. Molds and related components are 3D printed in PLA using FFF. The PET reinforcement is wound around the mold core, and the liquid-state silicone is poured into the mold. After ~2 minutes in the vacuum chamber and 10 hours of curing at room temperature, the unfinished actuator is removed from the mold and the mold core. This process is repeated in a second molding stage to add the internal elastomer layer.

**Movie S3**. Control

The hand fingers, controlled by the proposed ON-OFF control principle, keep the set bending angles for the index finger alone and the middle-ring-little fingers together. When the hand is not pneumatically actuated, it stays in a neutral pose. The ON-OFF controller regulates the airflow to the actuators, activating them and enabling smooth, human-like motion.
